# Incorporating genetics into the identification and treatment of Idiopathic Pulmonary Fibrosis

**DOI:** 10.1186/s12916-015-0434-0

**Published:** 2015-09-24

**Authors:** Susan K. Mathai, Ivana V. Yang, Marvin I. Schwarz, David A. Schwartz

**Affiliations:** Division of Pulmonary Sciences and Critical Care Medicine, Department of Medicine, University of Colorado Denver, Anschutz Medical Campus, 12631 East 17th Avenue, Aurora, CO 80045 USA

**Keywords:** *DSP*, Familial pulmonary fibrosis, Idiopathic pulmonary fibrosis, *MUC5B*, Mucin, Pulmonary fibrosis, *TERC*, *TERT*, Telomere

## Abstract

**Background:**

Idiopathic pulmonary fibrosis, the most common form of idiopathic interstitial pneumonia, is characterized by progressive, irreversible scarring of the lung parenchyma. Idiopathic pulmonary fibrosis has a poor prognosis, and there are no medical therapies available that have been shown to improve survival. It is usually sporadic, but there is evidence of familial clustering of pulmonary fibrosis, suggesting a genetic basis for this disease. More recently, studies have confirmed that specific genetic variants are associated with both familial and sporadic forms of pulmonary fibrosis.

**Discussion:**

Although there are common and rare genetic variants that have been associated with the risk of developing pulmonary fibrosis, the genotyping of patients is not a generally accepted strategy. Better understanding of the interplay between genetic risk and environmental exposure is likely needed to inform both treatment and disease prevention. Several identified disease-associated genetic variants have implications for disease progression and survival, but systematic studies of known genetic variants and their influence on therapeutic efficacy are lacking. Future investigations should focus on understanding phenotypic differences between patients carrying different risk alleles, and clinical studies should be designed to control for the influence of different genetic risk variants on patient outcomes.

**Summary:**

Inherited genetic factors play a significant role in the risk of developing pulmonary fibrosis. Future studies will be needed to characterize patient phenotypes and to understand how these genetic factors will influence clinical decision-making for both diagnosis and treatment of idiopathic pulmonary fibrosis.

## Background

Idiopathic pulmonary fibrosis (IPF), the most common idiopathic interstitial pneumonia (IIP), is characterized by progressive scarring of the lung parenchyma. The prognosis of IPF is poor, with a median survival from time of diagnosis of 3 years [1, 2]. The precise etiology of this disease has remained elusive despite decades of research. It is thought that IPF results from the aberrant behavior of injured alveolar epithelial cells, which in turn produce growth factors that induce proliferation of resident fibroblasts, recruitment of fibrocytes, and epithelial-to-mesenchymal transition [3]. This is thought to lead to the formation of interstitial fibroblastic foci (a structure unique to Usual Interstitial Pneumonia, the the histopathologic pattern of IPF, the accumulation of extracellular matrix, and lung remodeling [3]. Recent evidence has shown that there is an inherited risk of developing IPF, and specific genetic variants have been identified that are strongly associated with the disease.

Initial investigations distinguished between familial and sporadic forms of IPF, though there is increasing evidence that genetic risk factors play a significant role in both forms of the disease [4–6]. Although investigators continue to uncover genetic risk factors for disease and to probe their connections to IPF pathophysiology, the full clinical implications of these genetic discoveries remain unknown. Here, we briefly summarize the current knowledge regarding genetic risk and the development of IPF, describe how these genetic findings may influence the clinical management of patients with IPF, and suggest avenues for further investigation into the clinical implications of genetic risk in this disease.

### Focus on familial disease: early investigation into genetic risk and pulmonary fibrosis

Early evidence of inherited risk for the development of pulmonary fibrosis was based on twin studies and familial aggregation of cases [7–10]. Even though these early studies suggested an inherited risk, the first specific disease associated gene variants were identified after 2000 and included surfactant protein mutations among familial cases of pulmonary fibrosis [11–14], specifically in the genes for surfactant protein C (*SFTPC*) and *SFTPCA* [12, 14, 15]. There are also rare familial syndromes associated with pulmonary fibrosis, such as Hermansky–Pudlak syndrome. This disorder is caused by defects in intracellular protein trafficking, such as mutations in *AP3B1*, which are central to this genetically heterogeneous autosomal recessive disorder [16, 17].

Pulmonary fibrosis also occurs in dyskeratosis congenita, a syndrome characterized by aplastic anemia, myelodysplastic syndrome, skin hyperpigmentation, nail dystrophy, and pulmonary and liver fibrosis [18, 19]. There are a number of genetic mutations associated with dyskeratosis congenita, including mutations in dyskeratosis congenita 1 (*DKC1*), a gene involved in the stabilization of telomeres [18], as well as in other telomerase genes [19], pointing to telomeropathy as a potential underlying mechanism for fibrosis. Investigations of familial IPF cases and their kindred identified germline mutations in the telomerase genes telomerase reverse transcriptase (*TERT*) and telomerase RNA component (*TERC*) in up to one-sixth of pulmonary fibrosis families [19–21]. Importantly, *TERT* and *TERC* mutations were present in cases of both familial and sporadic IPF, and individuals with these mutations had shorter telomeres when compared to age-matched family members without mutations [22]. Recent studies by Cogan and colleagues describe rare variants in the genes encoding regulator of telomere elongation helicase 1 (*RTEL1*) and polyadenylation-specific ribonuclease deadenylation nuclease (*PARN*) associated with familial disease. These rare variants were found through exome-sequencing of familial interstitial pneumonia (FIP) cases [23, 24]. Patients with these variants had profound shortening of telomeres in peripheral blood mononuclear cells, though the mechanism by which loss of *PARN* affects telomere length is unknown. These newly described rare variants further point to telomere length’s being important in the pathogenesis of IPF [23, 24].

### Transition of focus from familial to sporadic disease

#### *MUC5B*

The previous studies focused on understanding genetic risk for disease conferred by rare variants by studying familial clustering of pulmonary fibrosis. However, in 2011, Seibold and colleagues found that common genetic variants were highly associated with familial as well as sporadic pulmonary fibrosis [6]. The authors used a genome-wide linkage analysis followed by sequencing to determine that a single nucleotide polymorphism (SNP) rs35705950 on the p-terminus of chromosome 11 is strongly associated with IPF as well as with FIP. FIP in this study was defined by the presence of two or more cases of definite or probable IIP within three generations of a family [6].

The common variant rs35705950 is found in the promoter region of the of mucin 5B (*MUC5B*) gene, which codes for a highly conserved region of the mucin promoter across primate species. Heterozygous (GT) and homozygous (TT) individuals had an odds ratio for developing disease of 6.8 and 20.8 for FIP, and 9.0 and 21.8 for IPF, respectively, demonstrating the strength of the SNP's association with disease development. Furthermore, an IPF diagnosis was associated with a more than 14-fold increase in *MUC5B* expression in the lung regardless of genotype, but the presence of the minor allele (T) at rs35705950 was associated with a 37.4-fold increase in gene expression even in unaffected individuals. MUC5B has also been found in honeycomb cysts, one of the hallmark pathologic findings of IPF [25].

This discovery was further validated in seven independent non-Hispanic white cohorts [4, 26–31], and the *MUC5B* promoter polymorphism remains the strongest and most replicated genetic risk factor for pulmonary fibrosis. In the initial study describing the association between rs35705950 and IPF, the minor allele frequency was 33.8 % in familial cases, 37.5 % in sporadic IPF cases, and 9.1 % in control subjects [6]. This highlights two important points: (1) the frequency of the risk allele is the same in familial and sporadic cases of IPF and (2) the frequency of the risk allele in the general non-Hispanic white control group implies interplay between genetic risk and environmental exposure in the development of IPF.

The importance of the rs35705950 variant in the pathogenesis of lung fibrosis was further illustrated by a recent study that examined the Framingham Heart Study population and found that the rs35705950 minor allele frequency was 10.5 %. After adjusting for covariates, the odds of radiographic interstitial lung abnormalities were 2.8 times greater for each copy of the rs35705950 minor allele. This study for the first time demonstrated a link between this polymorphism and radiographic interstitial lung abnormalities that could be considered “pre-fibrotic,” and also suggested that the rates of definite radiographic evidence of pulmonary fibrosis in individuals over 50 years of age may be 2 %, higher than what had been reported previously in the literature [1, 32]. These results from the Framingham Heart Study population support the notion that genetic information may be able to guide interventions to detect early fibrosis or pre-fibrotic lung lesions in asymptomatic individuals, suggesting a potential role for disease prevention, in addition to treatment, in the management of IPF [32].

The association of the *MUC5B* promoter polymorphism appears to be specific to pulmonary fibrosis. Cohorts with systemic sclerosis and interstitial lung disease [29, 33], asbestosis, sarcoidosis [27], acute lung injury or acute respiratory distress syndrome, chronic obstructive pulmonary disease, and asthma have failed to show strong associations between disease and genotype for this variant [34]. In addition, rs35705950 was a strong genetic risk factor for IPF in a Mexican population (odds ratio = 7.36, *P* = 0.0001), but was rare in Korean cases of IPF and was absent in Korean healthy controls [35]. Other studies found that the SNP had slightly higher prevalence among Japanese IPF cases (3.4 %) compared to healthy controls (0.8 %), and among Chinese IPF cases (3.3 %) compared to controls (0.7 %) [31, 36]. The prevalence of the *MUC5B* promoter SNP across different populations reflects disease prevalence in different racial or ethnic backgrounds: Caucasians appear to be at a higher risk of developing IPF than Hispanics and Asians while this disease is rare in populations of African descent [37]. Interestingly, the *MUC5B* polymorphism is not present in African populations [38]. Therefore, rs35705950 is likely to be important in some groups beyond the non-Hispanic white population for the development of IPF.

#### Other common variants and IPF

Genome-wide association studies (GWAS) have been performed in patients with one of the fibrotic IIPs (of which IPF is the most common) to identify additional variants that confer risk of disease. In 2013, Fingerlin and colleagues published a case–control GWAS in 1616 non-Hispanic white IIP patients and 4683 controls. This was supported by a replication study of 876 cases and 1890 controls [4]. This study confirmed the association between *TERT* at chromosome 5p15, *MUC5B* at 11p15, and the 3q26 region near *TERC,* but also identified seven new loci associated with disease, including *FAM13A* (4q22), *DSP* (6p24), *OBFC1* (10q24), *ATP11A* (13q34), *DPP9* (19p13), and chromosomal regions 7q22 and 15q14-15 [4]. These common variants associated with fibrotic IIP suggest that host defense (*MUC5B*, *ATP11A*), cell–cell adhesion (*DSP* and *DPP9*), and DNA repair (*TERT*, *TERC*, and *OBFC1*) may be important in disease pathogenesis [4, 34, 39]. These genetic loci, excluding rs35705950, account for approximately one-third of disease risk, emphasizing the importance of inherited genetic risk in disease pathogenesis [4, 34]. Furthermore, similar to the rs35705950 observations made by Seibold and colleagues, there were no substantial differences in odds ratios for disease between patients with familial and sporadic IPF, or between different forms of non-IPF IIP, suggesting that (1) the genetic risk factors for fibrotic IIPs are similar and that (2) familial and sporadic cases of IPF have similar genetic backgrounds.

A second GWAS performed in patients with IPF confirmed the association of the *MUC5B* promoter variant with IPF and also identified additional variants in Toll-interacting protein (*TOLLIP)* and signal peptide peptidase-like 2C (*SPPL2C)* as potential risk loci [28].

## Discussion: clinical implications of genetic discoveries in pulmonary fibrosis

### Phenotypic differences in carriers of the rare telomerase mutations

Identification of the constellation of findings (liver abnormalities, cytopenias, premature graying of the hair) consistent with rare genetic mutations in *TERT* or *TERC* mutations is critical, as these patients are at risk for bone marrow failure and cryptogenic liver cirrhosis [40]. Evidence suggests that in autosomal dominant forms of FIP caused by coding mutations in *TERT*, a unique form of genetic anticipation causes a shift from a pulmonary fibrosis predominant phenotype to one characterized by bone-marrow failure over successive generations [21]. Patients carrying *TERT* mutations have a poor prognosis with reduced life expectancy [41].

One of the few therapeutic options for patients with pulmonary fibrosis is lung transplantation. In the case of patients with *TERT* mutations, a small observational study suggests that complications of lung transplantation, such as renal failure, may be more common in IPF patients with telomerase mutations and/or shortened telomere syndrome [42], suggesting that genotyping could be important in determining transplant eligibility. This is particularly relevant in post-transplant patients who require significant immunosuppression because patients with these telomerase mutations experience increased rates of bone marrow suppression and medication-related complications [42], which may reflect their underlying diminished their underlying diminished bone marrow reserves. The authors caution that this observation has yet to be confirmed in larger cohorts of patients but suggest that careful consideration of the patients’ hematologic and hepatic status is warranted pre-transplantation [42].

### Survival differences based on genetic risk

Three observational studies have illustrated that common genetic variants associated with disease are also associated with differences in survival. In 2013, Noth and colleagues reported that several variants in *TOLLIP* were associated with IPF; however, carriers of the minor allele (G) at rs5743890 had decreased decreased risk of IPF but those with IPF who had this allele experienced increased mortality [28]. Another study published the same year described a survival advantage for individuals with the minor allele at rs35705950, the *MUC5B* promoter polymorphism strongly associated with disease [33]. Another functional SNP found in Toll-like receptor 3 (*TLR3*) has been associated with increased mortality and with accelerated disease progression in patients with IPF [43]. The mechanism for these observed differences in mortality remains unknown, but could be related to underlying differences in disease pathogenesis or in the clinical response to commonly prescribed therapies.

As numerous investigators have shown, various genetic variants, both rare and common, in telomere-related genes are associated with disease status [20, 23, 24]. Telomere length itself is also associated with transplant-free survival time for patients with IPF independent of age, sex, forced vital capacity, or diffusing capacity of carbon monoxide [44]. Additional studies are necessary to establish what the clinically relevant telomere length thresholds might be and how this measurement could function as an IPF biomarker or affect choice of therapy.

### Genotypes in the clinic and in clinical trials

**Fig. 1:**
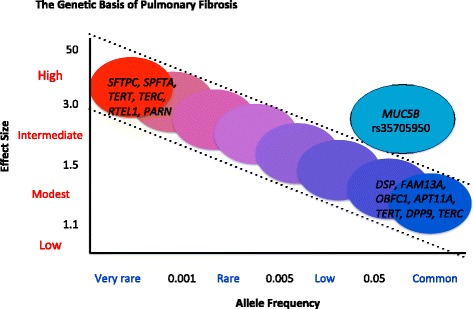
The genetic basis of idiopathic pulmonary fibrosis. This figure represents the spectrum of genetic variants known to confer risk for idiopathic pulmonary fibrosis and their relative allele frequency and effect size. Though rare variants have a low allele frequency, their effect size is profound, whereas common variants, though strongly statistically associated with disease, have a low effect size. The *MUC5B* promoter polymorphism rs35705950, though common, has a significant effect size. The low effect size of the common variants could suggest a strong gene-by-environment interaction in the overall risk of disease. (Adapted and reproduced with permission from Antonarakis et al., *Nature Rev Genetics* 2010, 11:380.) [53]

We suggest that further studies will continue to elucidate phenotypic differences between patients with IPF who have different disease-associated genetic variants. The clinical significance of these specific genetic variants remains unknown. Though strongly statistically associated with disease, the effect size of most common variants is small, whereas the effect size of rare variants is large (Fig. 1). The relationship between different common variants and their potential interaction with rare variants in disease pathogenesis will be an area of future investigation.

Because the clinical significance of these more common genotypes remains unknown, routine genotyping of individuals with IPF is not recommended. Furthermore, no evidence exists to suggest that genetic data should determine the selection of approved therapies for IPF, such as pirfenidone [45], nintedanib [46], or lung transplantation [47], for any individual patient. At this time, the specific treatment options for any given patient should be made on the basis of the published known risks and benefits of the medications, all of which have been studied independently of genotype [45, 46].

However, future investigations and clinical trials will need to take into account potential genotypic variation between different genotypes, especially as they likely affect the primary outcomes for clinical trials [48, 49]. Failure to control for genotypes, such as the *MUC5B* promoter polymorphism, would be tantamount to failing to control for other factors such as age, sex, and baseline lung function that are known to influence clinical outcomes. Post*-*hoc analysis of existing clinical trial data stratifying groups by the presence of common risk alleles might also generate intriguing hypotheses to be validated in prospective studies.

### Risk for family members

Given the unpredictable clinical course of IPF, its poor prognosis, and lack of available mortality-modifying medical treatment, it is important to identify individuals with early disease. Given the low prevalence of IPF, it is not a disease for which physicians routinely screen asymptomatic patients. However, the growing evidence for inherited disease risk may prompt the pulmonary community to reconsider the need to seek out patients for early diagnosis among at-risk populations.

In the case of FIP, it is known that first-degree relatives of patients with pulmonary fibrosis are at high risk of developing lung abnormalities, but the clinical significance of these abnormalities are unclear [8]. In 1986, Bitterman and colleagues studied family members of patients with autosomal-dominant FIP. They found that first-degree family members without clinically apparent disease had bronchoalveolar lavage fluid with increased inflammatory cells, but whether these individuals developed pulmonary fibrosis was not studied [8]. Twenty-seven years later, follow-up evaluation of two of these patients revealed interim development of radiographic evidence of pulmonary fibrosis, as well as symptomatic and measurable respiratory impairment [50]. Though this study was limited by its small sample size, it illustrates that alveolar inflammation in first-degree relatives of FIP patients can progress to overt pulmonary fibrosis and that these patients can experience a long duration of preclinical disease. More recently, extensive phenotyping of first-degree relatives of patients with FIP revealed evidence of dysfunction in pathways associated with the development of pulmonary fibrosis, including telomere shortening, endoplasmic reticulum stress, and elevated MUC5B levels [51]. These findings were observed in relatives with and without evidence of disease by high-resolution computed tomography or transbronchial lung biopsy, suggesting that these at-risk individuals have molecular abnormalities that precede symptoms or clinical detection. More than one-third of the at-risk subjects had histologically abnormal lung tissue, and 14.7 % had evidence of early interstitial lung disease [51]. Further observation will be required to determine the significance of these findings and of the results of the Framingham Heart study [32] with respect to identifying which asymptomatic subjects will progress to pulmonary fibrosis and whether early intervention prevents clinical worsening.

Although numerous studies have now shown that asymptomatic individuals who are at risk based on pedigree or who carry known risk alleles like the rs35705950 variant have higher rates of interstitial lung abnormalities [32, 51], there is no data to suggest that intervention is indicated. In part, this is due to the lack of data concerning the natural history of asymptomatic interstitial lung abnormalities. However, family members of patients with FIP should remain vigilant for the development for respiratory symptoms and should refrain from exposure to known environmental pulmonary toxins such as tobacco smoke [52].

## Summary

There is growing evidence that IPF is a disease in which genetic risk plays a central role. There are both common and rare variants that are associated with increased disease risk, and future studies will need to clarify the relationship between these variants and environmental exposures in the initiation and progression of IPF. The consideration of genetic risk factors in IPF will allow us to better phenotype the disease, because observational studies have shown that genotypes significantly affect clinical outcomes. A better understanding of genetic risk and its role in disease diagnosis will lead to detection of early asymptomatic cases and will allow us to personalize therapeutic choices based on inherited risk.

## Abbreviations

FIP: familial interstitial pneumonia
GWAS: genome-wide association studies
IIP: idiopathic interstitial pneumonia
IPF: idiopathic pulmonary fibrosis
SNP: single nucleotide polymorphism
